# Enzymatic Synthesis of Amino Acids Endcapped Polycaprolactone: A Green Route Towards Functional Polyesters

**DOI:** 10.3390/molecules23020290

**Published:** 2018-01-30

**Authors:** Stéphane W. Duchiron, Eric Pollet, Sébastien Givry, Luc Avérous

**Affiliations:** 1 BioTeam/ICPEES-ECPM, UMR CNRS 7515, Université de Strasbourg, 25 rue Becquerel, 67087 Strasbourg CEDEX 2, France; stephane.duchiron@etu.unistra.fr; 2 J. SOUFFLET S. A., Centre de Recherche et d’Innovation Soufflet—Division Biotechnologies, Quai du Général Sarail, 10402 Nogent sur Seine CEDEX 2, France; sgivry@soufflet.com

**Keywords:** enzymatic polymerization, caprolactone, amino acids, methionine, cysteine, ring opening polymerization, *Candida antarctica* lipase B, polyester functionalization

## Abstract

ε-caprolactone (CL) has been enzymatically polymerized using α-amino acids based on sulfur (methionine and cysteine) as (co-)initiators and immobilized lipase B of *Candida antarctica* (CALB) as biocatalyst. In-depth characterizations allowed determining the corresponding involved mechanisms and the polymers thermal properties. Two synthetic strategies were tested, a first one with direct polymerization of CL with the native amino acids and a second one involving the use of an amino acid with protected functional groups. The first route showed that mainly polycaprolactone (PCL) homopolymer could be obtained and highlighted the lack of reactivity of the unmodified amino acids due to poor solubility and affinity with the lipase active site. The second strategy based on protected cysteine showed higher monomer conversion, with the amino acids acting as (co-)initiators, but their insertion along the PCL chains remained limited to chain endcapping. These results thus showed the possibility to synthesize enzymatically polycaprolactone-based chains bearing amino acids units. Such cysteine endcapped PCL materials could then find application in the biomedical field. Indeed, subsequent functionalization of these polyesters with drugs or bioactive molecules can be obtained, by derivatization of the amino acids, after removal of the protecting group.

## 1. Introduction

During the last two decades, there has been a high focus in polymer for biomedical applications, for numerous purposes from drug delivery to tissue engineering [1,2]. All these fields require specific physical, chemical and biological properties. To reach such properties, several architectures have been developed from polymer chemistry by copolymerization, grafting or chemical functionalization [3,4,5]. There is a growing need for polymers with easily tunable properties which can combine different macromolecular architectures.

One of the most important properties required for most biomedical uses is the polymer biocompatibility [2], in connection with the non-toxicity of the degradation products and residual monomers [6]. Poly(ε-caprolactone) (PCL) is a well-known polyester that has been extensively studied in the last decades because of its non-toxicity, biocompatibility and bioresorbability [6,7,8,9,10,11,12]. PCL-based materials have been developed for numerous biomedical applications such as tissue engineering, wound dressing or single-use medical equipment [8,9,10]. For example, Park et al. demonstrated its great potential for bone-repair scaffolds [11] and several articles report its use for drug release and delivery systems [11,12].

Nowadays, the ring opening polymerization (ROP) of lactones by metal-based catalysts is the main way to obtain well-controlled polyester chains [13,14]. However, these residual catalysts may induce some toxicity (in the case of biomedical applications) [15], environmental pollution (in the case of compostability) and an increase in the polymer degradation kinetics [16] which could limit the material use. As potential alternatives, some common organocatalysts (like 4-dimethylaminopyridine, triflic acid or *N*-heterocyclic carbene) [17] show great potential for lactone ROP, but may also present some toxicity issues and drawbacks.

Enzymatic catalysis currently shows a great potential to substitute toxic metal-based catalysts or organocatalysts to limit the final toxicity and negative environmental impacts, in perfect agreement with a sustainable development and concepts of green chemistry [18]. By enzymatic catalysis, reactions can be performed under mild conditions (low temperature and pressure). Systems with high catalytic activity and very good reaction control of enantio-, chemo-, regio-, and stereo-selectivity can be expected. Owing to these advantages, enzymatic processes could provide precise control of the final polymer architectures, allowing the synthesis of polymers by a clean process, without by-products and with energy savings [19]. Enzymatic polymerization can thus be regarded as an environment-friendly synthetic process for polymeric materials, providing one of the best examples of “green polymer chemistry”. Among the vast choice of enzymes, lipases (E.C. 3.1.1.3) can be found in most organisms from microbial, plant and animal kingdoms [20]. They are serine hydrolase that catalyze ester bond cleavage in aqueous medium (physiological action is cleavage of triglyceride) and they are also able to catalyze esters bond formation in organic medium (reverse reaction) [21]. Currently, lipases seem to be the most efficient or at least the most known enzymes for polyester synthesis by enzymatic ring opening polymerization (eROP).

Lactones are one of the most widely studied monomers in eROP catalyzed by lipases [22], especially by lipase B from *Candida antarctica* (CALB) which is the most efficient enzyme for such reaction. Some studies have shown that large lactones with low ring strain are more compatible with lipases binding site than shorter ones with higher ring strain [23]. Interestingly, chemical ROP shows the inverse reactivity trend with monomer ring strain.

Because of the chemical similarity, other heterocycles based on sulfur, and to a lesser extent on nitrogen, have been studied. Indeed, if some works have been published on the eROP of thiolactones [24,25,26,27,28,29], the enzymatic synthesis of polyamides by ROP seems much more difficult and there are only a few works on the eROP of lactam. These studies show that lactam unit integration is limited. Besides, it is very difficult to obtain long polyamide chains because of huge stability of large lactam compared to β-lactam [30,31,32]. However, lipases could catalyze the synthesis of lactams from five to nine membered cycles, and the formation of cyclic amino acids dimers and trimers [33]. Some other strategies have been used to synthesize polyamides or polyesteramides such as polycondensation reaction catalyzed by lipases (e.g., synthesis of low molar mass poly(aspartic acid)) [34] or eROP catalyzed by proteases instead of lipases or cutinases which are the most commonly used enzymes [34,35,36,37]. Some authors also tried to modify enzymes by mutagenesis, aiming at improving polyamide production, but with mixed results so far [38].

However, polyesters containing peptides or amino acids are of great interest for pharmaceutical and biomedical applications such as drug carriers and controlled drug delivery systems or as resorbable implants and tissue engineering materials. Indeed, polyester functionalization with amino acids has been reported to improve the biomedical properties compared to the parent polymer [39,40]. Among the various benefits arising from the introduction of amino acids into polyester chains, one may cite the improved interactions with proteins, cells or genes as well as the possibility for subsequent functionalization with bioactive molecules or drugs [39,41]. Such polyester–amino acid or polyester–peptide conjugates can be obtained by chemical modification of previously formed polyester chains, or by using a functionalized ester monomer but a more elegant way consists in using peptide or amino acids as (macro-)initiators, for instance, for the ring opening polymerization of lactones [42,43]. Various natural amino acids have been tested and reported in the literature for their ability to initiate the ring opening polymerization of ε-caprolactone (CL) or lactide, in bulk, without the use of metal-based catalysts [43,44,45,46]. However, the literature on the chemical polymerization of ε-caprolactone initiated by amino acids remains limited and, to the best of our knowledge, the enzyme-catalyzed ROP of CL initiated by natural amino acids has never been reported.

In the present work, we thus report assays of two distinct synthetic routes to enzymatically synthesize functional polyesters based on ε-caprolactone (CL) and two selected natural amino acids. Cysteine (Cys) and methionine (Met) have been selected since their respective side groups present adequate chemical functions: (i) a thiol for cysteine, which allows subsequent branching, grafting or cross-linking; and (ii) a thioether for methionine that could allow a control of the polymer thermal resistance, for example by sulfonation of the thioether [47]. A protection–unprotection approach of the chemical groups has been used and then two different strategies were tested: (i) preliminary tests based on direct polymerization, using methionine and cysteine amino acids; and (ii) an indirect approach, involving protected cysteine. The macromolecular architectures and the mechanisms to obtain the corresponding polymers have been analyzed through different techniques. The main goal of this study is to synthesize amino acids containing polyesters, and to compare and draw conclusions on the relative reactivity of acid, ester, amine and thiol functions, by enzymatic catalysis.

## 2. Results and Discussion

**Figure 1:**
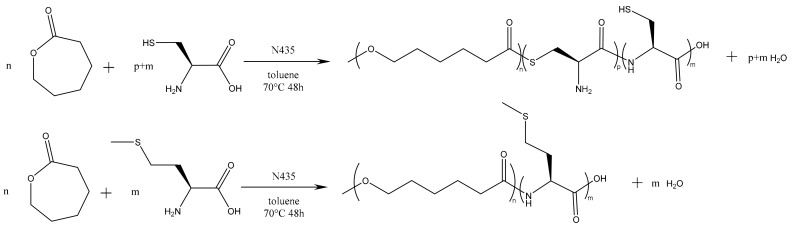
Preliminary direct approach for the polymerization of CL with unmodified amino acids.

In a preliminary approach, we tested the direct enzyme-catalyzed polymerization of CL with the unmodified amino acids, as shown in Figure 1.

Main results of the corresponding syntheses are summarized in Table 1, where the first line corresponds to the blank test without amino acid, the second to the fifth lines correspond to CL polymerization with cysteine and the last four to CL polymerization with methionine. These experiments allow to compare the two amino acids (AA) and the effect of the AA feed content.

Table 1 clearly shows that addition of small amounts of amino acids in the reaction medium does not lead to significant differences on the resulting polymers. The number average molar masses (Mn) are quite constant, ranging from 10,500 to 11,500 g·mol^−1^ with cysteine and from 10,500 to 11,200 g·mol^−1^ with methionine. These values are slightly lower than for the blank test that shows a molar mass around 15,000 g·mol^−1^. This could be due to the initiation by amino acids or more likely by the water molecules brought by the amino acids that are very hygroscopic compounds. It is noteworthy that these values are given as polystyrene (PS) standards and are thus largely overestimated. The real Mn values are likely around half those presented here [46] and are in good agreement with those reported for amino acids initiated polymerization of CL in bulk [43,44,45,46]. One can also notice the relatively high dispersity (Đ), around 2, which could mean that there are two different initiation steps and/or competitive transesterification reactions. As reported in a previous work, the acrylic resin-immobilized form of CALB (N435) is an efficient transesterification catalyst [48]. This high dispersity is also linked to a long reaction time (48 h) compared to the more conventional 24 h often used for eROP of CL [49]. Such a high dispersity could also be explained by an initiation either by the amine of methionine or cysteine or by the thiol of cysteine, both being in competition with the conventional water initiation. Indeed, if initiation involves amine, thiol and hydroxyl (water) groups then their different reactivity may lead to different initiation rate constants that will generate different chains populations that could contribute to a higher dispersity. For the sake of comparison, dispersity values ranging from 1.3 to 1.9 have been reported for amino acid initiated PCL [43,44,45,46]. However, the occurrence of numerous transesterification reactions is more likely the main reason for the high dispersity observed here.

From the TGA results, one can observe a non-negligible weight loss before the main degradation peak (Td at around 400 °C) mainly due to water (below 150 °C) and residual CL monomer (below 250 °C) losses. These molecules have been chemically identified by FTIR analysis of the evolved gas from the degradation (Figure S1).

A representative DSC thermogram is presented in Figure S2 but all products display similar pattern. Indeed, one can notice from the DSC results (Table 1) that thermal properties seem not to be affected by the addition of amino acid during the synthesis and these values are very close to those expected and observed for PCL homopolymer. For example, melting temperature (Tm) ranges between 50 °C and 52 °C, which is comparable to, or slightly below, the melting temperature of PCL of such molar mass [43,50]. Only the crystallinity (Χ) shows some variations, without clear trend, but these values are very sensitive to the limits set for the peak integration and thus to the melting peak width. Nevertheless, these values are in good agreement with the crystallinity reported for PCL obtained from arginine-based macro-initiators [43]. In addition, all products show a double melting peak, which is also consistent with the thermal behavior of PCL chains of similar molar mass. This is due to crystals imperfections and some difficulties for the polymer to crystallize when molar masses are limited [50]. In the same way, degradation temperatures (Td around 400 °C) are very close to the one of neat PCL. These results tend to demonstrate that there is no, or a very limited, insertion of amino acids in the PCL polymer chain.

This has been confirmed by the ^1^H- and ^13^C-NMR analyses displaying only the characteristic peaks of PCL chains (Figure S3), highlighting that mainly PCL homopolymers have been obtained. The presence of residual CL has also been confirmed by ^1^H-NMR spectroscopy (Figure S3a), with an estimated content ranging from 1% to 3% after the polymer precipitation recovery step, depending on the AA feed content. The highest value is obtained for the reaction involving 10 mol % Cys which also corresponds to the lowest CL monomer conversion. Such presence is somewhat expected since our precipitation protocol in cold methanol (−78 °C) is done to recover the smallest polymer chains but is also able to precipitate CL. One can notice that, initial amino acids do not appear on products analysis. They are probably precipitated at the end of the reaction when chloroform is added and then eliminated by filtration together with the enzyme.

**Figure 2:**
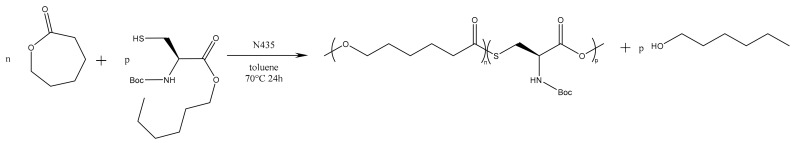
Second polymerization strategy based on the use of *N*-Boc protected cysteine hexyl ester.

The results of these preliminary tests show that the insertion of amino acid units in the polymer chain has not been clearly established and thus the corresponding products were not considered for further characterization. In a previously published study, Sobczak et al. claimed a successful initiation with both methionine and cysteine (at a content of ca. 2 mol %) and thus their incorporation in PCL chains but without giving any proof of such insertion [45]. In our case, it is assumed that the limited insertion of amino acids and their lack of reactivity are mainly due to their poor solubility in CL and toluene but that could also be due to a poor affinity with the lipase binding site. To improve the amino acid incorporation in the chain and to increase the solubility of the amino acid in the reaction medium, *N*-Boc cysteine hexyl ester (*N*-Boc Cys HE) (Figure 2) has been used. Indeed, tertiobutyl and hexyl group drastically increase the solubility of amino acids in organic solvent [51]. Besides, compared to the native carboxylic acid, hexyl ester could make the monomer closer to the lipase natural substrates (i.e., fatty esters) and could thus increase the monomer affinity with enzyme binding site, leading to higher reactivity.

However, since the first experiments did not show any reactivity of the thioether function of methionine, and since *N*-protected methionine would have only one reactive group left, making its polymerization impossible, this second strategy has been used only on cysteine derivatives.

Results of the polymerization reactions carried out on the modified cysteine are summarized in Table 2. At first, one can notice a significant decrease of the molar masses with the increase in amino acid feed content. This is consistent with the results reported by Liu et al. and Sobczak et al. showing a global decrease of the PCL molar mass with increasing amount of amino acid [44,45]. Besides, such decrease is not surprising since there are four different potential initiating species. Two of them are initially present in the reaction medium (residual water and *N*-Boc Cys HE) and the others are produced during the polymerization reaction (hexanol from transesterification of *N*-Boc Cys HE and water produced by reaction on thiol function of *N*-Boc Cys HE). Among these four potential initiators, three of them thus show an increasing content with the increment of the amino acid feed content. One can also notice that, for the 1% *N*-Boc Cys HE feed content, the final molar mass is higher than in preliminary step and close to the blank test (see Table 1). This is likely due to the lower hygroscopicity of the protected amino acid compared to native one.

According to these results, one can notice that there are on average only 1 to 1.3 amino acid units per chain. This means that the amino acid reacts mainly as initiator or as chain ending species. This is consistent with the results reported in the literature for similar systems and the amino acid content is also in agreement with contents reported for creatine endcapped PCL [45]. This low content in incorporated amino acid is also confirmed by TGA analyses with the mass loss below 250 °C which corresponds to the loss of the Boc protecting group, the residual water and the unreacted monomers as shown by the FT-IR coupling (Figure S1).

**Figure 3:**
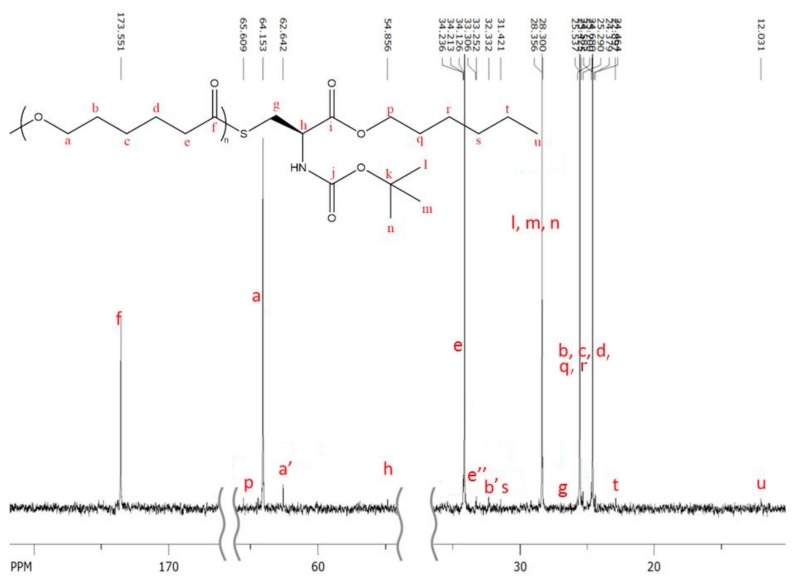
^13^C-NMR of polymerization product between CL and 10 mol % of *N*-Boc Cys HE.

The ^13^C-NMR characterization shows interesting pattern (Figure 3). Most of the characteristic peaks of protected amino acid could be easily identified except for the three peaks corresponding to the carbonyl of Boc protecting group, to the quaternary carbon of the tertiobutyl group and to the carbonyl of hexyl ester function. The absence of these peaks is not surprising because of the usually weak response of sp^4^ carbons in ^13^C-NMR and the small amount of amino acid in the polymers (with a maximum of 1.9 mol %, Table 2).

Interestingly, one could also notice that the signals of hexyl ester are always observed in the polymer which could mean that at least part of *N*-Boc Cys HE did not react on its hexyl ester function or that hexanol released by transesterification of *N*-Boc Cys HE initiated an eROP side reaction. This second possibility is more probable due to the well-known ability of N435 to catalyze transesterification reactions.

One can also notice on the ^13^C-NMR spectrum several small peaks at 63 ppm and between 30 ppm and 34 ppm (see assignment on Figure 3) with a’ and b’ corresponding to alcohol chain-end and e” corresponding to thioester linkage.

These results seem to indicate that several distinct populations of polymer chains could be formed due to some side reactions, such as hexanol initiation, but also due to the multiple reactive sites on the *N*-Boc Cys HE monomer.

**Figure 4:**
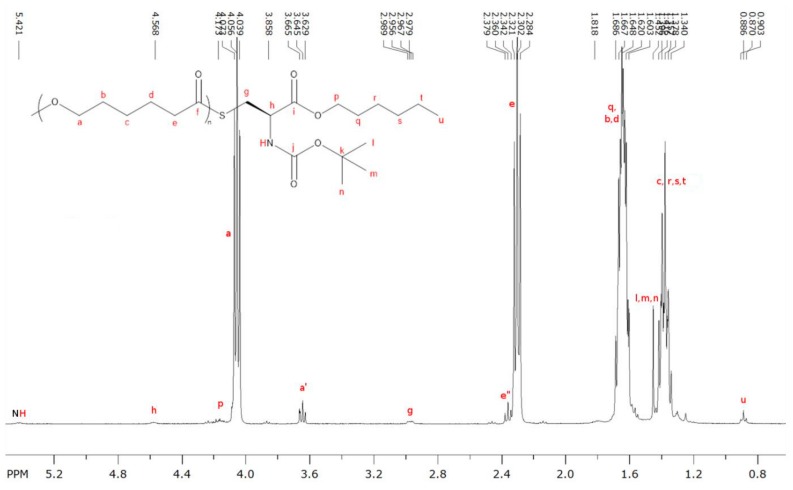
^1^H-NMR of polymerization product between CL and 10 mol % of *N*-Boc Cys HE.

^1^H-NMR spectra of the products obtained from CL polymerization with *N*-Boc Cys HE (Figure 4, Figures S4a, S5 and S6 for 10%, 1%, 2% and 5% amino acid feed content, respectively) show significantly different patterns than those observed with the first polymerization strategy.

From such spectrum, one can clearly see that the polymer chains are mainly composed of CL units. Here again low intensity signals corresponding to alcohol chain-end (a′) and to the thioester linkage (e′′) can be identified. However, residual monomer content in the products cannot be determined precisely due to peaks overlapping at 4.1–4.2 ppm but is more likely lower than 5%. Besides, all characteristic peaks of *N*-Boc Cys HE can be clearly identified on the ^1^H-NMR spectrum (Figure 4). This includes the hexyl ester signals corresponding to the chain population initiated by a thiol function and to the possible population initiated by hexanol resulting from the transesterification of cysteine hexyl ester. However, these analyses did not allow us to determine which reaction is more likely and to conclude on this specific point.

**Figure 5:**
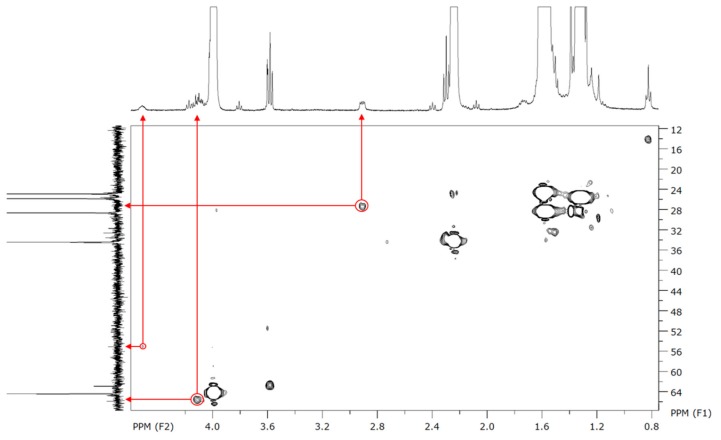
HSQC (^1^H–^13^C) 2D NMR of polymerization product between CL and 10 mol % of *N*-Boc Cysteine HE.

To characterize more precisely those polymers, 2D NMR experiments were performed. Despite some absent (or too low intensity) signals on the ^13^C-NMR, the proposed peaks assignment has been confirmed by HSQC (^1^H–^13^C), two-dimensional NMR experiment which directly shows the bonded protons and carbons. Figure 5 shows the confirmation of our assignment for the low intensity peaks in ^13^C-NMR for carbons g and h, which make coupling spots at, respectively, 27 and 55 ppm.

**Figure 6:**
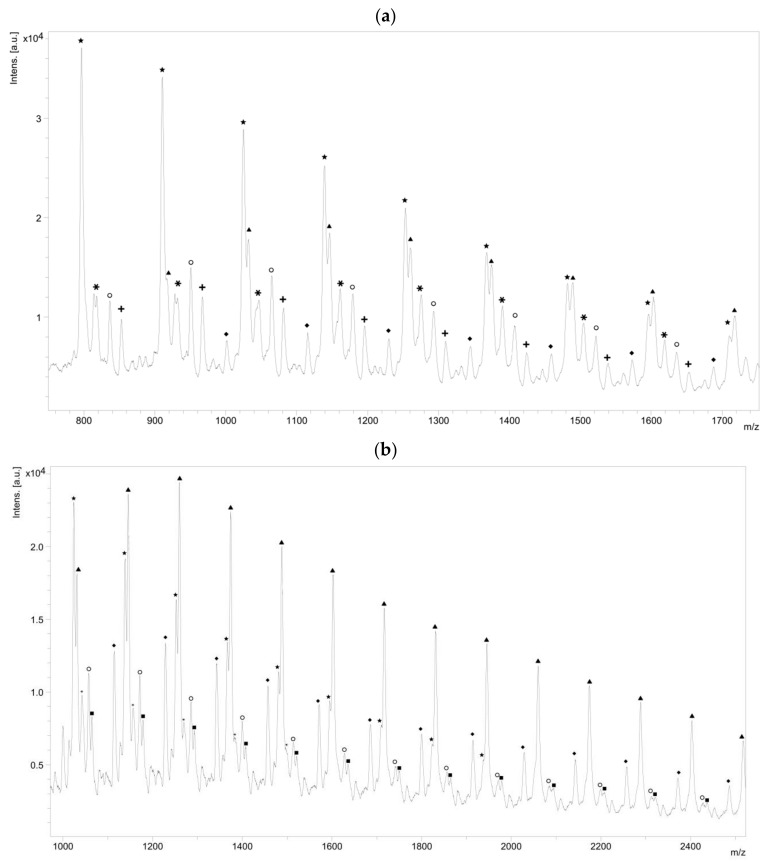
MALDI-ToF MS spectrum of polymerization product between CL and: (**a**) 2 mol %; and (**b**) 10 mol % of *N*-Boc Cys HE.

The NMR spectroscopy highlighted the possible presence of multiple polymer chain populations but, despite the deep analysis of the spectra, their different chemical structures remain only partially elucidated. Then, to clarify such complex polymer chains populations and structures, MALDI-ToF mass spectrometry analyses of the polymers of CL with *N*-Boc Cys HE were performed. Two examples of the resulting mass spectra are shown in Figure 6a,b, corresponding, respectively, to the polymerization products with 2% and 10% of *N*-Boc cysteine hexyl ester in the feed. The mass spectrum of the product obtained with 1% of *N*-Boc Cys HE in the feed is available in Figure S4b.

One can see in Figure 6a that there are at least six different distributions of polymer chains. These analyses also show that the *m*/*z* value of each peak logically verifies Equation (1).

(1) $$m/z=\;M_{Na}-M_{H}+nM_{CL}+M_{chain-ends}$$

**Figure 7:**
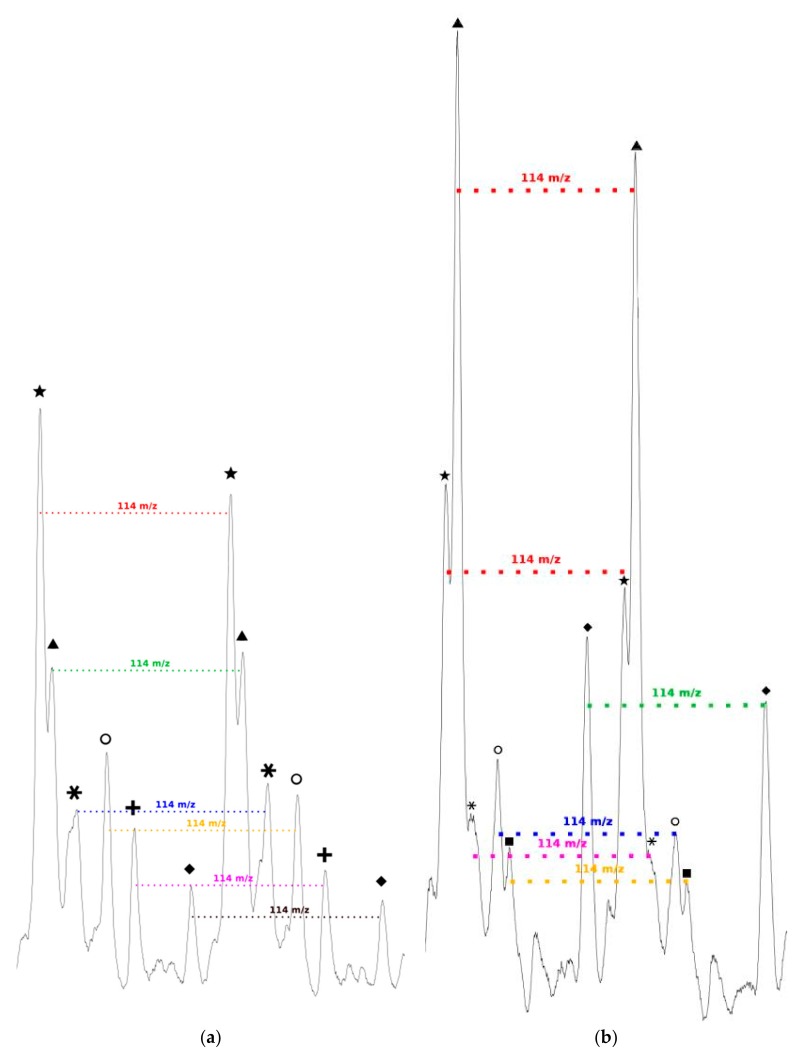
Interval between the successive peaks of a same polymer chain distribution for the polymerization of CL with: (**a**) 2 mol %; and (**b**) 10 mol % of *N*-Boc Cys HE.

We can also notice in Figure 7a that, for all distributions, the interval between two successive peaks of a same distribution is 114 *m*/*z*, which exactly corresponds to one CL unit. This result validates our previous hypothesis made from the amino acid content and confirms that there is roughly only one amino acid per polymer chain. This is also in perfect agreement with MALDI-ToF results reported for PCL chains initiated by phenylalanine or arginine [45,46]. Regarding the *m*/*z* values distribution, one can observe a significant difference with the Mn values determined from SEC. This can be explained by the overestimation arising from the values given as PS standards but also from underestimated values given by MS analyses due to higher response usually observed for the lower molar mass compared to the longer chains.

A detailed analysis of these mass spectroscopy spectra allows to identify and propose the different types of chain-ends and/or polymer structure corresponding to each distribution. The main proposed structures and mechanisms for polymers obtained with 2 and 10 mol % of *N*-Boc Cys HE in the feed are summarized in Table 3.

Concerning the products obtained with 2 mol % of *N*-Boc Cys HE in the feed, as expected, the main distribution corresponds to chains initiated by the thiol function of cysteine and this initiation seems to be more important than the one from residual water. This could easily be explained by the very low water content since we minimize it for each product and at each step. One can also notice chains termination by an amino acid due to transesterification reaction involving the hexyl ester. Interestingly, this termination seems to become more important for longer polymer chains. That could indicate that after a first oligomerization/polymerization step, the chain growth is then mainly made by transesterification and chain coupling. As supposed from the NMR results, there is a chain distribution initiated by hexanol resulting from transesterification of the hexyl ester amino acid (Figure 2). From the MALDI-ToF mass spectra, one can also identify the presence of cyclic PCL chains which have already been reported in the literature [45,46,52]. All these chains populations resulting from transesterification reactions have already been reported for amino acids endcapped PCL [45,46].

**Figure 8:**
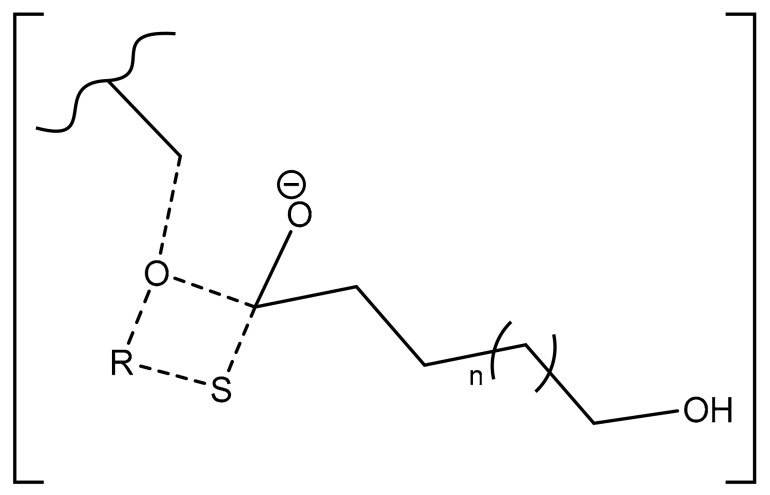
Potential intermediate formed in the enzymatic polymerization deacylation step that could lead to either a thioester- or an ester-initiated chain.

Surprisingly, there is another distribution with a rather unexpected chain-end. According to the *m*/*z* values, a putative structure involving a *N*-Boc Serine hexyl ester (*N*-Boc Ser HE) is proposed. This hypothesized structure could originate from a substitution of the sulfur atom of the modified cysteine amino acid by an oxygen (from the serine residue of the active site) during the deacylation step of the reaction. A potential intermediate that could explain such mechanism is proposed (Figure 8) but there is neither experimental data nor existing literature references to support this statement.

However, from the low peak intensity in the mass spectra, it seems that such substitution would be of very low occurrence and could explain why such products were not identified by NMR spectroscopy (also because their characteristic peaks could be masked by other signals).

Interestingly, for high amino acid contents in the feed (10 mol %), the MALDI-ToF mass spectrum is slightly different (Figure 6b). Indeed, six main distributions are also observed in this case but with two main differences in the identified structures (Table 3). One can first notice the absence of chains initiated by water molecules. This could be explained by the higher amount of amino acid which makes water molecules negligible compared to the others available initiators such as the thiol function of cysteine and hexanol released by transesterification of *N*-Boc Cys HE. As already mentioned, the occurrence of such transesterification reactions, that are promoted and catalyzed by the lipase, had been previously reported and observed from MALDI ToF spectra for similar systems [45,46]. As a second difference, one can also notice a surprising chain population that seems to be terminated by a cysteine. This distribution could result from a termination reaction by transesterification with unprotected cysteine hexyl ester. The latter could originate from a protected amino acid that would have been deprotected during the synthesis of *N*-Boc Cys HE. Indeed, amino acid esterification reaction is performed in acidic medium and this could weaken, and possibly remove, the *N*-Boc protecting group. However, according to the literature, lipases are unlikely to catalyze such Boc deprotection [53]. However, this chain distribution seems to be the least important.

One can also notice from the peaks intensities that the relative amounts of the various distributions are also different for polymers synthesized with larger quantity of amino acid in the feed. For instance, for the low amino acid content, the main distribution seems to be the chains initiated by the cysteine thiol whereas for higher feed content in amino acid, the chain terminated by transesterification of the hexyl ester seems to be major one. This latter behavior could be explained by the lower molar mass obtained when the feed content in protected amino acid is increased. As a result, protected amino acid chain ends are in greater ratio. The occurrence of lipase catalyzed transesterification reactions affecting these chain ends is thus statistically higher. It was also noticed that for longer chains, the sulfur substitution seems to be more important while this had not been observed for low amino acid feed content. However, as mentioned before, this trend is unexplained and the proposed mechanism and structure remain uncertain.

As seen in Figure 7b, and as already observed for the low amino acid content, for all distributions the interval between two successive peaks of a same distribution is 114 *m*/*z*, which corresponds exactly to one CL unit. This result confirms that, even if the amino acid content in the feed is increased, there is only around one amino acid unit incorporated in a polymer chain.

## 3. Materials and Methods

### 3.1. Materials

ε-Caprolactone (CL) was purchased from Sigma Aldrich (St. Louis, MO, USA) and distilled over CaH_2_ under reduced pressure before each use. Methionine (Met) and cysteine (Cys) amino acids as well as Novozym^®^435 (N435, Novozymes, Bagsværd, Denmark), the acrylic resin-immobilized form of *Candida antarctica* lipase B (CALB), were purchased from Sigma Aldrich (St. Louis, MO, USA) and used after freeze drying. Anhydrous toluene was freshly distilled over sodium under nitrogen atmosphere prior each use. Hexanol was freshly distilled on molecular sieve under vacuum prior use. Other chemicals (*N*-Boc cysteine, *N*-Boc methionine, 4 Å molecular sieve, *p*-toluenesulfonic acid, and NaHCO_3_) and solvents (methanol, diethyl ether, ethanol, chloroform, and dichloromethane) were purchased from Sigma Aldrich (St. Louis, MO, USA) and used without further purification or drying.

### 3.2. N-Boc Cysteine Hexyl Ester (N-Boc Cys HE) Synthesis

Typically, 1 g of *N*-Boc cysteine (4.5 mmol) was added in a round bottom flask with 20 equivalents of freshly distilled hexanol (11.4 mL; 90 mmol) under argon atmosphere. Then 2 mol % of *p*-toluenesulfonic acid were added to the reaction flask and the system was capped with a distillation bridge. Then the system was heated to 140 °C for 3 h, few droplets of water were collected by the distillation bridge. After cooling to 20 °C, the reaction medium was neutralized by NaHCO_3_. After filtration, the organic phase was diluted in diethyl ether and washed several times with water. Then, residual alcohol and solvent were distilled to produce about 1.05 g (76% of yield) of a slightly yellow oil. ^1^H-NMR (CDCl_3_; 400 MHz): δ = 0.87–0.90 ppm (t, 3H, *H*_3_C-CH2-); δ = 1.25–1.39 ppm (m, 6H, CH_3_-C_3_*H*_6_-CH_2_CH_2_CO-); δ = 1.45 ppm (s, 9H, tertiobutyl); δ = 1.53–1.70 ppm (m, 2H, C_4_H_6_-C*H*_2_CH_2_O-); δ = 2.91–3.12 ppm (m, 2H, HS-C*H*_2_CH); δ = 4.15–4.27 ppm(m, 2H, CH_2_-C*H*_2_O); δ = 4.35 ppm (s, 1H, NH); δ = 4.48–4.62 ppm (m, 1H, HS-CH_2_-C*H*-).

### 3.3. Enzymatic Polymerization Setup

All reactions were carried out in dry toluene (2 mL), at 70 °C. For that, 3.95 mmol of CL (0.9017 g) and the adequate quantity of amino acid in molar equivalent (from 40 μmol to 0.395 mmol; e.g., for cysteine from 4.8 mg to 47.8 mg for 1% to 10 mol %, respectively) and 4 Å molecular sieve (0.05 g) were introduced into a previously dried Schlenck tube under an inert dry argon atmosphere. The tube was immediately capped with a rubber septum and then immersed in a heated oil bath at 70 °C. Toluene was transferred with a syringe through the rubber septum cap. A predetermined amount of N435 catalyst (50 mg) was quickly introduced in the tube under an inert dry argon atmosphere. The tube was immediately capped with a rubber septum. The enzyme addition marked the beginning (t0) of the polymerization. Reactions were terminated by dissolving the reaction mixture into chloroform and removing the catalyst by filtration. Part of the solvent in the filtrate was then stripped by rotary evaporation at 35 °C. The polymer in the resulting concentrated solution was precipitated in cold methanol (in a dry ice-ethanol bath at approximately −70°C) to ensure also the recovery of the shortest chains. The precipitated polymer was recovered by filtration and dried overnight at 30 °C under vacuum. For the polymerization involving *N*-Boc cysteine hexyl ester, similar procedure than for eROP of unprotected amino acids and CL was performed.

### 3.4. Characterization Techniques

NMR analyses were performed on a Bruker Ascend™ 400 spectrometer (Bruker, Wissembourg, France) operating at 400.13 MHz and 100.62 MHz for ^1^H and ^13^C NMR, respectively. Spectra were obtained by performing at least 64 scans for ^1^H, 1024 scans for ^13^C, 8 scans for COSY and 8 scans for HSQC analyses. 1D and 2D NMR spectra were exploited with SpinWork 4.1 freeware software (Dr K. Marat, University of Manitoba, Winnipeg, Canada). All samples were prepared in deuterated chloroform with typically 8 mg for ^1^H and 15 mg for ^13^C and 2D NMR analyses. The determination of *N*-Boc cysteine hexyl ester content in the polymer was calculated according to Equation (2):

(2) $$N-\mathrm{Boc}\;\mathrm{cysteine}\;\mathrm{hexyl}\;\mathrm{ester}\;\left(\%\right)=100\;x\;\frac{I_{Boc}/9}{\;\left(I_{Boc}/9\right)+\left(I_{PCL}+I_{PCL\;endchain}\right)/2}$$

using the integration of the singlet at 1.45 ppm, corresponding to the 9 protons of Boc tertiobutyl group, compared to the sum of the integrals of the two triplets at 3.65 and 4.06 ppm corresponding to the ROC*H*_2_R in poly(ε-caprolactone) chain-end and main chain units, respectively. For the syntheses with unprotected cysteine, the residual CL monomer content in the final product was calculated from the ratio of the integrals of the CL and PCL characteristic peaks (respectively, at 4.1 and 4.0 ppm).

Size exclusion chromatography (SEC) measurements were performed in chloroform (HPLC grade) in a Shimadzu liquid chromatograph equipped with a LC-10AD isocratic pump, a DGU-14A degasser, a SIL-10AD automated injector, a CTO-10A thermostated oven with a 5 μm PLGel Guard column, two PL-gel 5 μm MIXED-C and a 5 μm 100 Å 300 mm-columns, and three online detectors: a Shimadzu RID-10A refractive index detector, a Wyatt Technologies MiniDAWN 3-angle-light scattering detector and a Shimadzu SPD-M10A diode array (UV) detector. Samples were dissolved in chloroform (concentration 4 mg·mL^−1^) and filtered through a 0.45 μm PTFE membrane. For all analyses the injection volume was 100 μL, the flow rate was 0.8 mL·min^−1^ and the oven temperature was set at 25 °C. The given molar masses and dispersity were calculated from a calibration with polystyrene standards in RI detection. Refractive index increment values (dn/dc) were measured by injecting a known concentration of polymer and assuming 100% mass recovery from the system.

MALDI-ToF analysis starts with the samples preparation. Matrix solutions were freshly prepared with Super DHB (9:1 mixture of 2,5-dihydroxybenzoic acid and 2-hydroxy-5-methoxybenzoic acid, from Sigma Aldrich (St. Louis, MO, USA)) which was dissolved till saturation in a H_2_O/CH_3_CN/HCOOH (50%/50%/1%) solution. Typically, a 1:1 mixture of the sample solution in CH_2_Cl_2_ was mixed with the matrix solution and 1 μL of the resulting mixture was deposited on the stainless steel plate. Mass spectra were acquired on a time-of-flight mass spectrometer (MALDI-ToF-ToF Autoflex II ToF-ToF, Bruker Daltonics, Bremen, Germany) equipped with a nitrogen laser (λ = 337 nm). An external multi-point calibration was carried out before each measurement using the singly charged peaks of a standard peptide mixture (0.4 μM, in water acidified with 1% HCOOH). Scan accumulation and data processing were performed with FlexAnalysis 3.0 software (Bruker Daltonics, Bremen, Germany).

The thermal stability and degradation of the samples was investigated by a thermal gravimetric analyzer (TGA) coupled with a FTIR for evolved gas analysis. TGA measurements were conducted under dry air (at a flow rate of 75 mL·min^−1^) using a Hi-Res TGA Q5000 apparatus from TA Instruments (New Castle, DE, USA). The samples (5–9 mg placed in a platinum pan) were heated up to 450 °C at 5 °C·min^−1^. FTIR spectra were recorded on a Nicolet 380 (Thermo Electron Corporation, Waltham, MA, USA) by performing 16 scans with 4 cm^−1^ resolution.

DSC measurement were performed on a TA Q200 DSC (New Castle, DE, USA). in sealed aluminum pan, typically on about 1 mg of purified sample, in a heat-cool-heat cycle at 10 and 5 °C·min^−1^ for heating and cooling, respectively. Results were exploited using TA Universal Analysis software (New Castle, DE, USA). The crystallinity of the samples was determined using the neat PCL common value of 139.5 J·g^−1^ for $∆H_{m}^{0}$.

## 4. Conclusions

This work investigated the enzymatic polymerization of ε-caprolactone with unmodified or modified sulfur-containing amino acids (cysteine and methionine). Two strategies were tested, a first one based on a direct polymerization of CL with the native amino acids and a second route involving the use of amino acids with protected functional groups. Even though lipases are catalysts that work well at aqueous–organic interfaces and which could then be efficient for syntheses in heterogeneous systems, the results from our first polymerization strategy did not show any significant reactivity of the amino acids. This lack of reactivity has been ascribed to a poor solubility of the amino acids in the reaction medium and to their low affinity with the lipase active site.

A second synthetic strategy based on the use of *N*-Boc and hexyl ester protected amino acid was thus established to enhance the solubility and improve the affinity with the lipase binding site. The significant conversion observed for the protected amino acids confirms that the solubility of these compounds and their affinity with the lipase binding site are key parameters for an efficient initiation of the CL enzymatic polymerization. This strategy produced various polymer chains populations that are mainly PCL homopolymer chains with distinct initiators and/or chain-ends, as highlighted by NMR and MALDI-ToF analyses. Each of the identified polymer chains distributions led us to formulate hypotheses on the mechanism of such enzymatic polymerization that once controlled could pave the way for the synthesis of new functionalized biocompatible polymers with potentially tunable macromolecular architectures and properties. Indeed, polyesters containing amino acids or peptides sequences are promising materials for biomedical applications such as controlled drug delivery systems or tissue engineering materials [39,40,41].

As far as cysteine-functionalized polymers are concerned, despite the different polymer backbones and synthesis strategies reported in the literature, some of the obtained materials have been described to form micelles showing enhanced cell adhesion or improved drug release properties [54,55]. In addition, the thiol group, which is present in some of the chains populations obtained with our strategy, and the carboxyl group can also be used for the grafting or conjugation of peptides sequences, antibodies, drugs or nanoparticles to obtain biomaterials with improved properties [56,57]. Regarding the polymer backbone functionalization using the thiol group, peptide conjugation using thiol-ene chemistry and native chemical ligation have been reported as efficient methods [56,57,58].

Thus, our polymerization strategy based on the use of modified amino acid opens perspectives for subsequent functionalization of the polymer chain by simply removing the protecting group that will make available a functional moiety on the polymer backbone. Then, further works would consist in investigating the polymer functionalization after deprotection and the potential biomedical applications of such cysteine-endcapped PCL chains.

## Figures and Tables

**Table 1:** Results obtained from the direct polymerization of ε-caprolactone (CL) with the two unmodified amino acids (methionine and cysteine).

Amino Acid	AA Feed Content (mol %)	Mn ^1^ (g/mol)	Đ ^1^	Tm (°C)	Tc (°C)	Χ (%)	Td ^2^ (°C)	η ^3^ (%)
-	0	15 000	1.7	54	34	59	400	95
Cys	1	11 300	2.1	51	36	52	401	92
Cys	2	11 200	2.1	51	32	51	399	90
Cys	5	11 500	2.2	52	32	47	400	85
Cys	10	10 500	2.1	52	36	46	399	86
Met	1	11 200	2.2	52	36	45	395	93
Met	2	11 000	2.2	50	35	49	398	89
Met	5	10 800	2.2	52	36	46	394	87
Met	10	10 500	2.1	51	35	55	396	84

^1^ Values determined by SEC and given as PS standards; ^2^ main degradation temperature at maximum degradation rate; ^3^ final yield obtained after vacuum drying.

**Table 2:** Results obtained from the polymerization of CL with *N*-Boc cysteine hexyl ester.

AA Feed Content (mol %)	Mn ^1^ (g/mol)	Đ ^1^	Tm (°C)	Χ (%)	Td ^2^ (°C)	Mass Loss at 250 °C (%)	η ^3^ (%)	AA Final Content ^4^ (%)
1	14 300	2.3	54	54	405	1.1	96	0.7
2	11 400	2.5	54	54	404	1.4	93	0.8
5	8 000	1.6	51	61	402	2.0	94	1.6
10	6 500	1.6	52	54	405	3.3	96	1.9

^1^ Values determined by SEC and given as PS standards; ^2^ main degradation temperature at maximum degradation rate; ^3^ final yield obtained after vacuum drying; ^4^ values determined by NMR.

**Table 3:** Proposed structures and mechanisms for CL polymerization with *N*-Boc Cys HE.

Marker	Structure	Supposed Mechanism
★	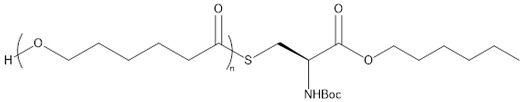	initiated by thiol of *N*-Boc Cys HE
○	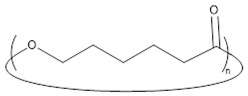	terminated by chain cyclization
▲	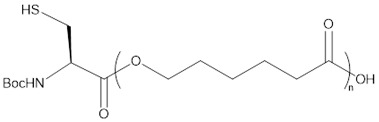	initiated by water and ending by transesterification of *N*-Boc Cys HE
*	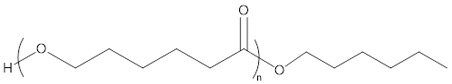	initiated by hexanol
+	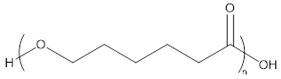	initiated by residual water
♦	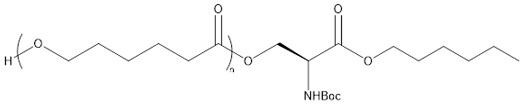	initiated by *N*-Boc Ser HE resulting from substitution of S by O in the binding site during the deacylation step
▪	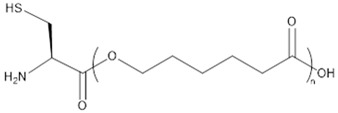	initiated by water and ending by transesterification of cysteine hexyl ester

## Supplementary Materials

Supplementary materials are available online.
