# Supplementary material for: Enzymatic Synthesis of Amino Acids Endcapped Polycaprolactone: A Green Route Towards Functional Polyesters
Source: Molecules. 2018 Jan 30;23(2):290. doi: 10.3390/molecules23020290 (PMC6017777; doi:10.3390/molecules23020290)
Supplement: Supplementary file 1 [file molecules-23-00290-s001.pdf]

Enzymatic Synthesis of Amino Acids Endcapped Polycaprolactone: A Green Route towards Functional Polyesters

Stéphane W. Duchiron [1], Eric Pollet [1,*], Sébastien Givry [2] and Luc Avérous [1,*]

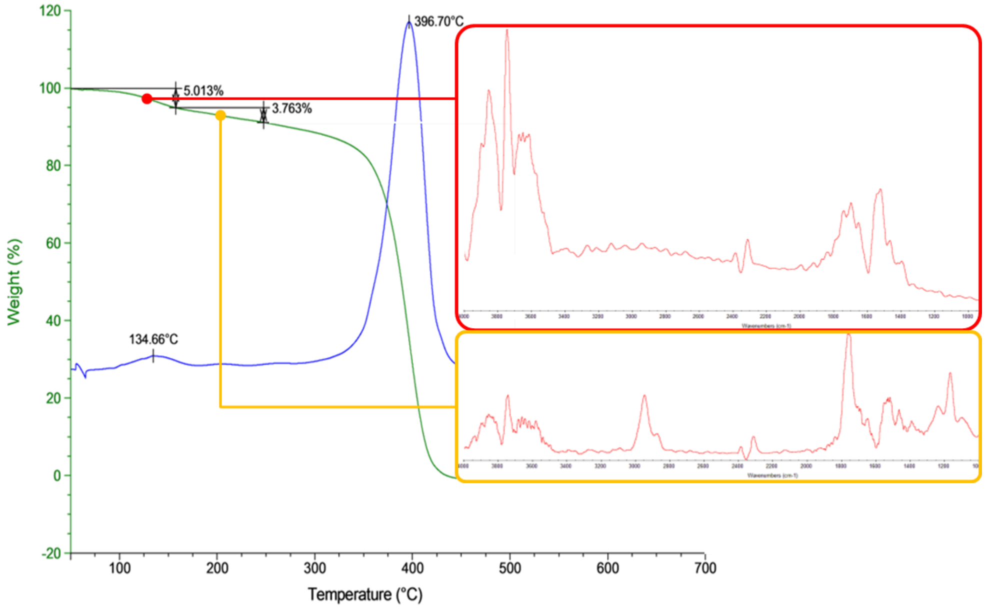

Figure S1. TGA-FTIR coupling analysis of polymerization product between CL and unprotected amino acid (10 mol% of Met in the feed) presenting the polymer main degradation (at ca. 400 °C). Red inlay (top) shows FTIR spectrum of evolved gas containing mainly water moisture. Yellow inlay (bottom) shows FTIR spectrum of evolved gas containing mainly CL residual monomer.

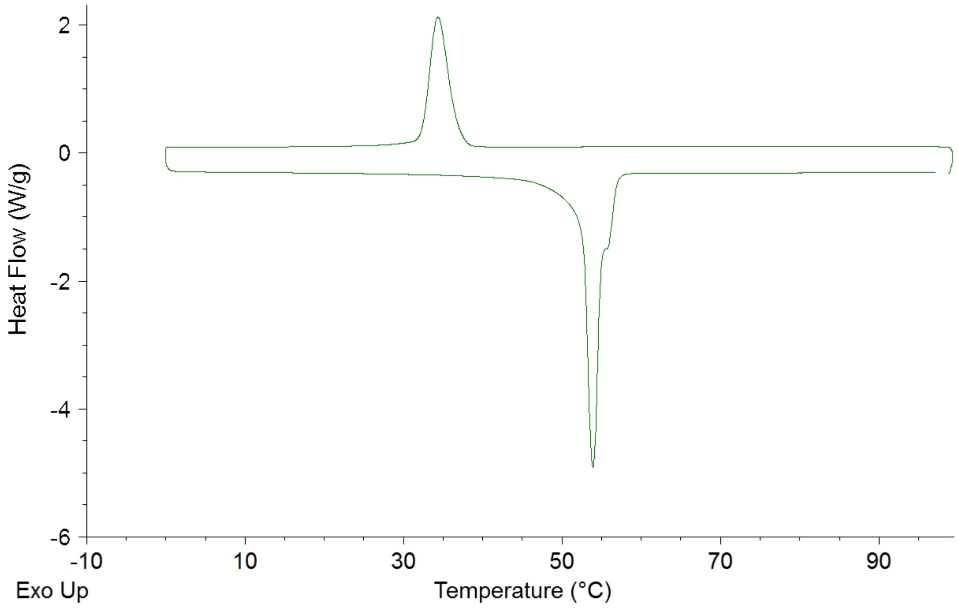

Figure S2. Representative DSC thermogram of polymerization product between CL and 2 mol% of N-Boc Cys HE (cooling scan followed by second heating scan).

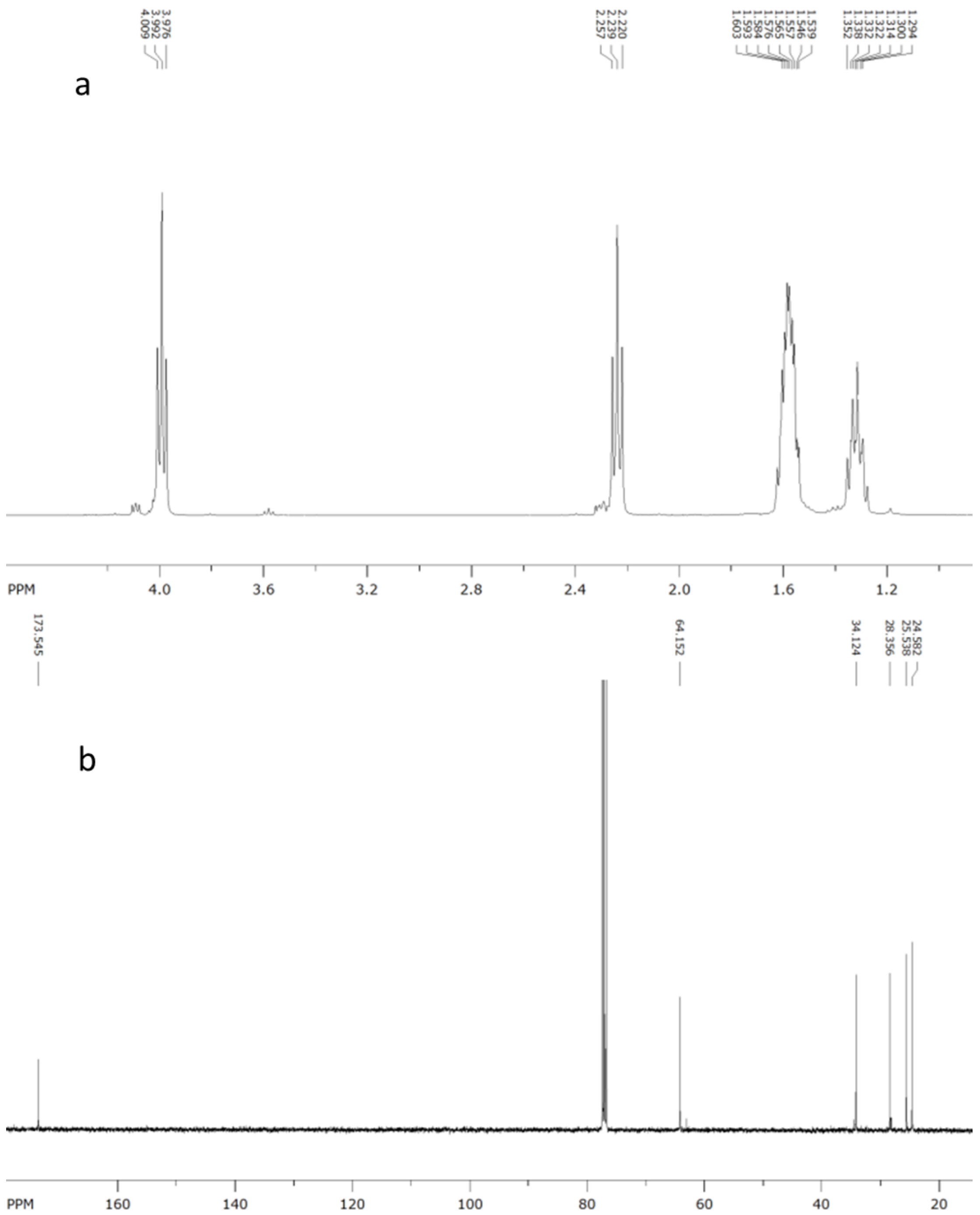

Figure S3. (**a**) ^{1}H NMR and (**b**) ^{13}C NMR typical spectra of polymerization product between CL and unprotected amino acid (example of 10 mol% of Cys in the feed).

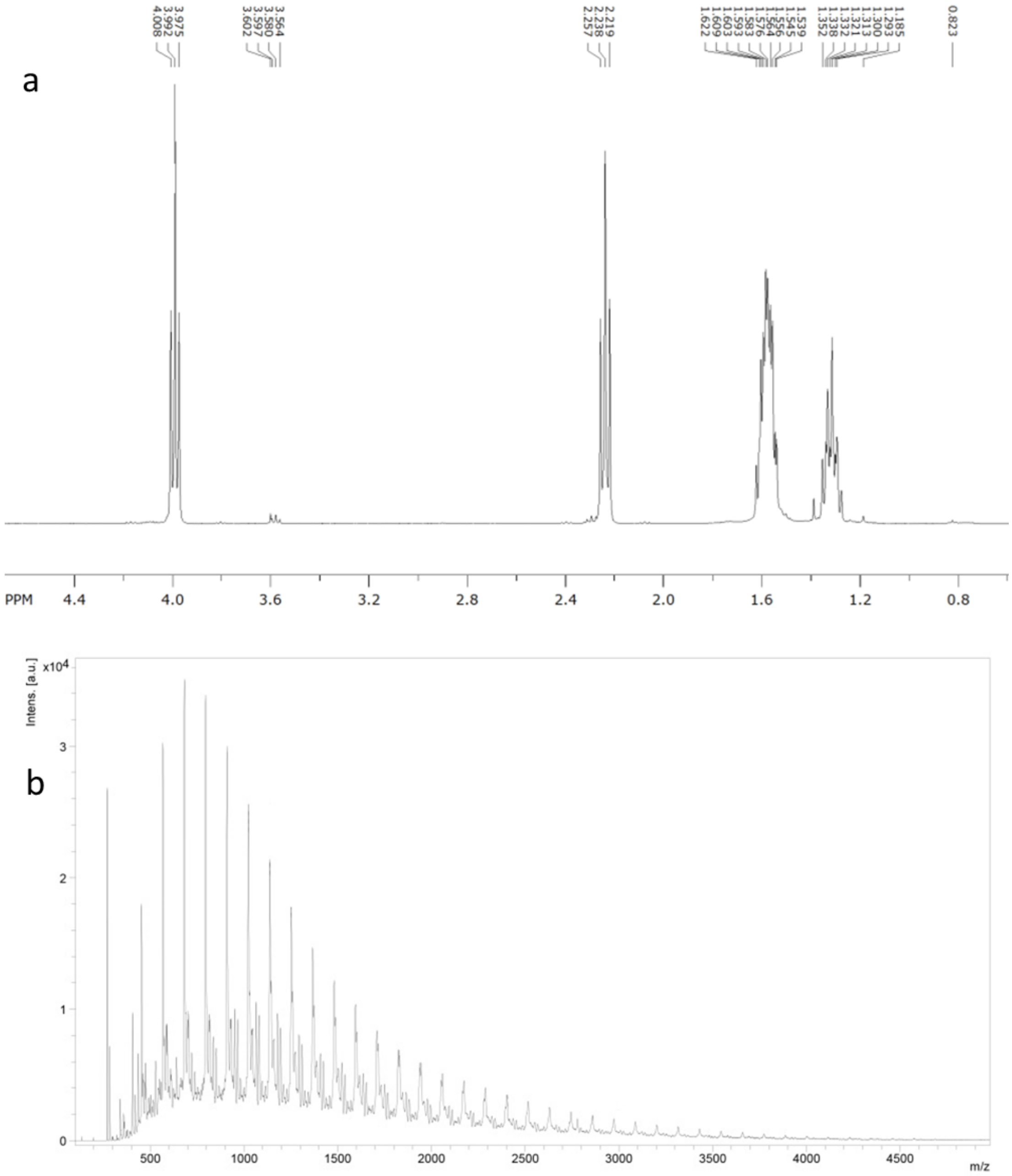

Figure S4. (a) ^{1}H NMR and (**b**) MALDI-ToF spectra of polymerization product between CL and 1 mol% of N-Boc Cys HE.

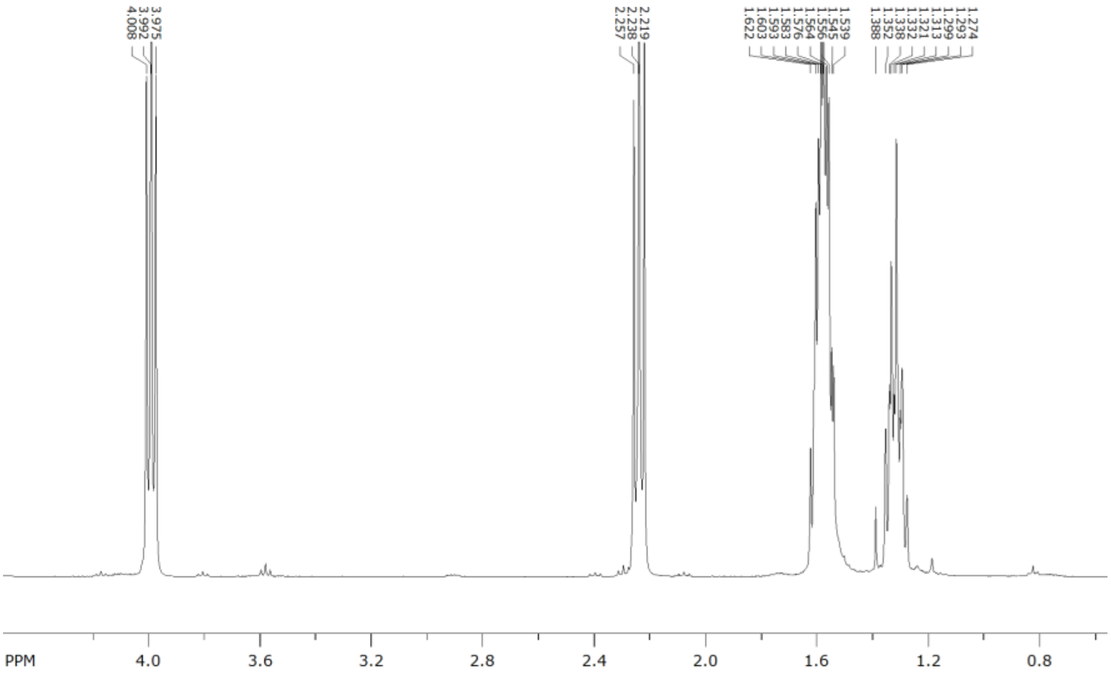

Figure S5. ^{1}H NMR spectrum of polymerization product between CL and 2 mol% of N-Boc Cys HE.

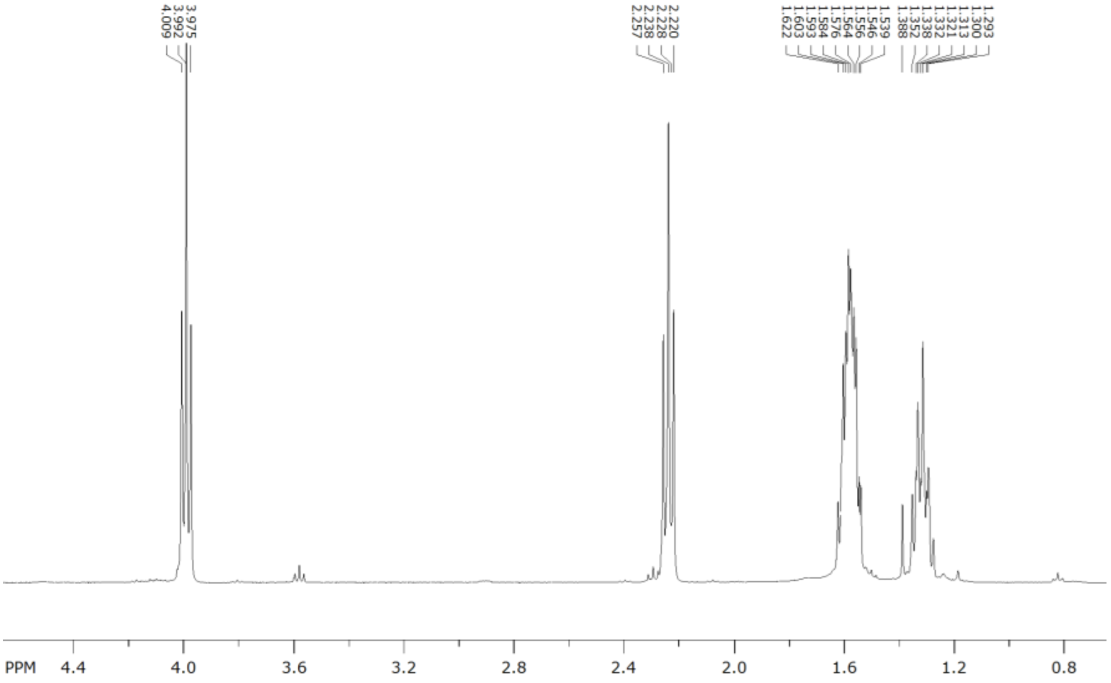

Figure S6. ^{1}H NMR spectrum of polymerization product between CL and 5 mol% of N-Boc Cys HE.
